# Pyrotinib-assisted whole brain radiotherapy alleviates HER2-positive advanced breast cancer and brain metastases: a prospective study in patients

**DOI:** 10.3389/fneur.2025.1439028

**Published:** 2025-02-14

**Authors:** Fulin Zhou, Cui Zhang, Mingyuan He, Yu Ren, Hongshuang Yue, Zhihua Tan, Shaolin Zhang, Yong Li, Shu Liu

**Affiliations:** ^1^Department of Clinical Medicine, Guizhou Medical University, Guiyang, China; ^2^Department of Breast Surgery, Guiyang Maternal and Child Health Care Hospital, Guiyang, China; ^3^Zunyi Medical University, Zunyi, China; ^4^Department of Oncology, The Affiliated Hospital of Guizhou Medical University, Guiyang, China; ^5^Department of Breast Surgery, The Affiliated Hospital of Guizhou Medical University, Guiyang, China; ^6^Department of Oncology, Guizhou Provincial People’s Hospital, Guiyang, China

**Keywords:** pyrotinib, breast cancer, whole brain radiotherapy, human epidermal growth factor receptor-2, brain metastasis

## Abstract

**Purpose:**

The whole-brain radiotherapy (WRBT)-based therapeutic efficacy is often limited against human epidermal growth factor receptor 2 (HER2+)-positive advanced breast cancer (BC) and brain metastases (BM), requiring more effective treatment options. This prospective study evaluates the effectiveness and safety of combining WBRT with pyrotinib in patients with HER2+ advanced BC and BM.

**Methods:**

The enrolled patients (*n* = 26, from April 2019 to March 2022) were divided into two treatment groups. Group 1 (p-WBRT) received pyrotinib initially, followed by subsequent WBRT. Group 2 (WBRT-p) received WBRT concurrently with pyrotinib. The intracranial progression-free survival (iPFS) was determined.

**Results:**

In the WBRT-p group (*n* = 11), the median iPFS was recorded as 25.0 months (95% CI, 15.3–34.7), while the overall survival (OS) rates in 1–4 years were 100, 54.5, 9.1, and 0%, respectively. The intracranial objective response rate (iORR) and intracranial clinical benefit rate (iCBR) were 63.6 and 90.9%, respectively. In the p-WBRT group (*n* = 15), the median iPFS was around 22.0 months (95% CI, 4.3–39.7), and the OS rates in 1–4 years were 100, 53.3, 33.3, and 6.7%, respectively. The iORR and iCBR values were 66.7 and 80.0%, respectively. Notably, no significant differences in iPFS, OS, iORR, and iCBR were observed between treatment groups. Although some instances of adverse events, such as vomiting and reduced white blood cells and neutrophil counts, were evident, these adverse events were grades 1–3.

**Conclusion:**

WBRT combined with pyrotinib exhibited exceptional tolerability, showing long iPFS in patients with HER2+ advanced BC and BM.

## 1 Introduction

Breast cancer (BC) has become one of the most dreadful and widespread cancer types in women, accounting for numerous cases globally. Around 20% of BC cases are characterized as HER2-positive (HER2+), which is associated with higher rates of recurrence and metastasis than its counterpart (1, 2). Notably, HER2+ patients are at an increased risk of developing metastases in the brain (BM) (3). Despite the advancements in BC treatments, the incidence and mortality rates of BM have been increasing. Previous research indicated that, even after receiving anti-HER2 treatment based on trastuzumab, pertuzumab, and lapatinib, 30–50% of patients with HER2+ metastatic BC still experience BM (4). Accordingly, BM has been increasingly recognized as a primary cause of death in HER2+ patients, significantly impacting their overall quality of life and mortality rates (5, 6). Therefore, there is an urgent need to develop efficient treatment options to enhance survival rates.

Currently, various treatment methods for BM in BC patients involve localized and systemic therapeutic modalities. Among them, radiotherapy is one of the most commonly employed approaches as a localized therapy. Accordingly, WBRT (Whole-brain radiotherapy) is often considered an effective treatment for individuals with multiple brain lesions, particularly when there are more than 4 lesions. However, the decision between WBRT and stereotactic radiosurgery (SRS) has evolved, especially with phase II data supporting the use of SRS for up to 10 lesions, as recommended by NCCN guidelines (7). While WBRT remains a key treatment modality, its effectiveness can be limited by factors such as the extent of disease dissemination and patient-specific health conditions. The QUARTZ study highlighted that for patients with extensive extracranial disease or those who are otherwise quite ill, WBRT may not offer significant survival benefits. However, this is a small subset of patients, and WBRT is generally effective in managing brain metastases in those with a better overall health status (8). In contrast, systemic therapies, particularly those targeting HER2, have shown promise in crossing the blood–brain barrier and providing additional intracranial control (9–11). Nevertheless, these therapies are not necessarily superior in all cases, and their efficacy must be evaluated within the context of each patient’s disease extent and overall health.

Pyrotinib, an orally administered irreversible pan-ErbB receptor TKI, effectively targets HER1, HER2, and HER4 (12). Previous research demonstrated the stability, safety, and well-tolerated pyrotinib in patients with advanced HER2+ BC (13, 14). In August 2018, the National Medical Products Administration approved the utilization of pyrotinib in combination with capecitabine against advanced or metastatic BC with HER2+ (15). In a case, the phase III PHENIX study considerably observed that, in women with HER2+ metastatic BC, the combination of capecitabine and pyrotinib increased the median progression-free survival (PFS; 11.1 months *vs.* 4.1 months) by 7.0 months, improving the objective response rate (ORR; 68.6% *vs.* 16.0%) compared to capecitabine alone. Moreover, patients with BM experienced a longer PFS of 6.9 months as opposed to 4.2 months (16). In another case, a phase 2 study, known as PERMEATE, demonstrated that pyrotinib + capecitabine effectively targeted both intracranial and extracranial lesions in patients with BM that are HER2+, particularly in patients who had not previously received radiation therapy. Notably, the combination therapy yielded a high intracranial ORR of 74.6% (95% CI: 61.6–85.0%) and a substantial 11.3-month PFS benefit (95% CI: 7.7–14.6) (17). In this study, we focused on a cohort of HER2+ advanced BC patients with brain metastases who had an ECOG performance status of 0–2 and a minimum life expectancy of 12 months. Our study demonstrated that combining pyrotinib with WBRT—regardless of timing—can result in promising outcomes for patients with HER2+ advanced breast cancer and brain metastases. This suggests that pyrotinib, when used in conjunction with WBRT, could be an effective treatment strategy.

## 2 Materials and methods

### 2.1 Study design

**Figure 1:**
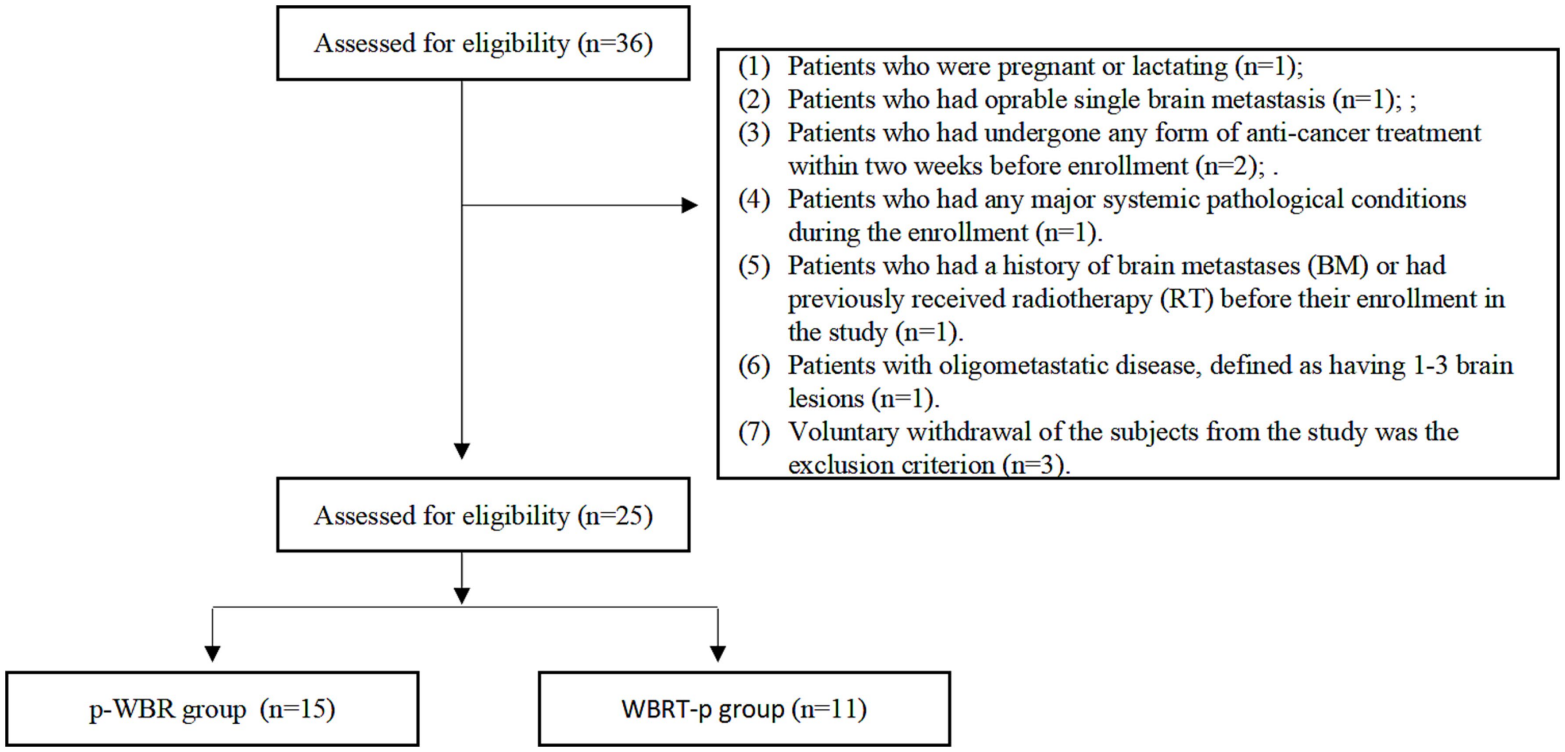
Flow chart of study inclusion criteria.

For this prospective study, patients diagnosed with advanced HER2+ BC and BM between April 2019 and March 2022 were enrolled (Figure 1). It should be noted that the study was conducted at three medical institutions, namely Guizhou Provincial People’s Hospital, Affiliated Hospital of Zunyi Medical University, and Guizhou Cancer Hospital. Following the Chinese laws, regulations, and the Helsinki Declaration guidelines, the study received authorization from the ethics committee at each participating facility. Moreover, all the enrolled patients had provided written informed consent prior to their involvement in the study.

### 2.2 Patient population (inclusion and exclusion criteria)

The inclusion criteria for the eligible patients for enrollment encompassed the following aspects: (1) Female patients who were aged ≥18 years; (2) Patients with Eastern Cooperative Oncology Group (ECOG) performance status ranging from 0 to 2; (3) The diagnosis of BC with HER2+ subtype was confirmed through pathological examination, either by an immunohistochemistry score of 3+ or positive results obtained from fluorescence *in situ* hybridization (FISH); (4) Histological or cytological inspection, as well as imaging examinations, confirming the presence of BM condition; (5) Patients with the existence of at least one detectable brain lesion larger than 10 mm. Notably, this was considered as per the criteria outlined in the Response Evaluation Criteria In Solid Tumors, version 1.1; (6) Patients with a minimum life expectancy of 12 months; (7) Patients with sufficient hematological, hepatic, renal, and cardiac functional data. On the other hand, the exclusion criteria for the enrollment were set as follows: (1) Patients who were pregnant or lactating; (2) Patients who had operable single brain metastasis; (3) Patients who had undergone any form of anti-cancer treatment within two weeks before enrollment. (4) Patients who had any major systemic pathological conditions during the enrollment. (5) Patients who had a history of brain metastases (BM) or had previously received radiotherapy (RT) before their enrollment in the study. (6) Patients with oligometastatic disease, defined as having 1–3 brain lesions, were not included in this study. All eligible patients were screened for inclusion, and those with severe underlying conditions or contraindications were excluded prior to enrollment.

### 2.3 Patient groups and treatment procedure

In this prospective study, the implementation of WBRT was based on the detection of brain metastases accompanied by clinical symptoms such as headaches, vomiting, seizures, optic nerve compression, or impaired vision. In such cases, WBRT is considered an essential treatment option for managing extensive brain metastases. All patients were comprehensively evaluated before determining the necessity of WBRT.

**Figure 2:**
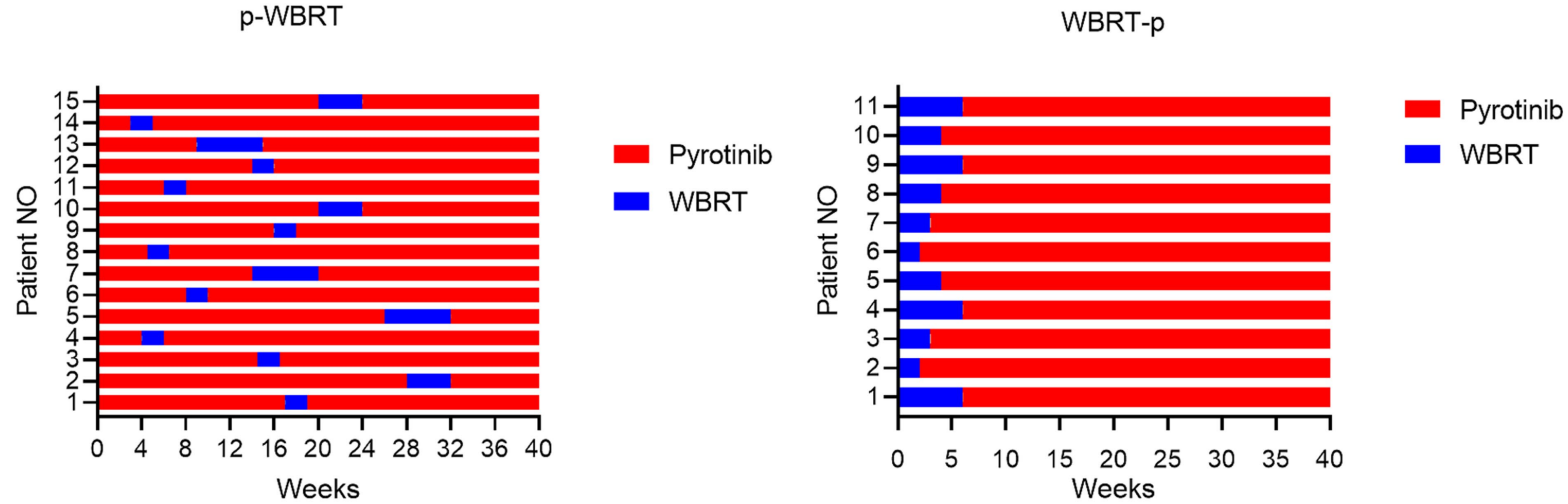
The first 40 weeks of treatment course for the p-WBRT and WBRT-p group were illustrated. All patients continued to received pyrotinib until the end of intracranial PFS.

Patients diagnosed with HER2-positive advanced BC and BM were assigned to two distinct treatment groups, based on the sequence of administration of pyrotinib and whole-brain radiotherapy (WBRT) (Figure 2):

Group 1 (p-WBRT): In this group, patients received pyrotinib first for a duration of 2 to 28 weeks (400 mg single dose orally per day) before initiating WBRT. The implementation of WBRT depended on the development of clinical symptoms of brain metastases. Pyrotinib treatment continued concurrently with WBRT. WBRT was delivered over a fixed regime with a total dose of 30 in 10 fractions in 2 weeks or 40 Gy in 20 fractions in 4 weeks. Some patients received additional local WBRT, with a total dose of 60 Gy in 6 weeks. Following WBRT, patients continued to received pyrotinib until the end of intracranial PFS (iPFS, the interval between the start of WBRT treatment and the advancement of intracranial disease or death from any cause).

Group 2 (WBRT-p): In this group, patients initially received a fixed regime with a total dose of 30 in 10 fractions in 2 weeks or 40 Gy in 20 fractions in 4 weeks. Some patients received additional local WBRT, with a total dose of 60 Gy in 6 weeks. All patients received concurrent pyrotinib until the end of intracranial PFS.

Moreover, the initial dosage and any necessary dose reductions, interruptions, therapy cessation, and combination methods were chosen at the discretion of the clinicians based on individual clinical symptoms. Notably, the WBRT procedure was performed in 10 fractions at a dosage of 30 Gy. The clinical follow-up evaluations were conducted every 2–3 weeks throughout the treatment period. Finally, the imaging examinations were performed every 3–6 weeks (equivalent to 1–2 drug cycles) in adherence to standard clinical guidelines.

### 2.4 Endpoints

After treatment, the performance efficacy, i.e., the main outcome of the designed combinatorial regimen, was determined based on the intracranial PFS (iPFS, the interval between the start of treatment and the advancement of intracranial disease or death from any cause). In addition to iPFS, the secondary endpoints included iORR, intracranial clinical benefit rate (iCBR), overall survival (OS) in 1–5 years, and treatment-related adverse events (TRAEs). However, the percentage of patients who experienced either a partial or complete intracranial response (iORR) was defined (PR). Patients with intracranial CR, PR, or SD ≥6 months were classified as iCBR. The term OS refers to the period between the start of therapy and the death of any cause. The TRAEs were evaluated based on Version 5.0 of the National Cancer Institute’s Common Terminology Criteria for Adverse Events.

### 2.5 Statistical analysis

To assess patient characteristics related to continuous data, the median value and range were determined, while categorical variables were presented as frequencies and percentages. The iPFS and OS values were calculated using the Kaplan–Meier (KM) technique, allowing us to compare the outcomes between different groups through the utilization of the log-rank test. To conduct the statistical analysis, SPSS version 26.0 was employed, while R 4.1.1 was employed for data visualization purposes. The statistical significance was set at a two-tailed *p*-value of less than 0.05.

## 3 Results

### 3.1 Patient characteristics

After eligibility evaluation, a total of 26 patients admitted between April 2019 and March 2022 were enrolled (11 in the WBRT-p treatment groups and 15 in the p-WBRT treatment group). Among them, a total of 10 patients (*n* = 10, 38.5%) showed hormone receptor-positive cancer disease. In addition to BM condition, some patients (*n* = 2, 7.7%) displayed bone and/or soft tissue metastases, visceral metastases (*n* = 7, 26.9%), and both visceral and non-visceral (bone and/or soft tissue) metastases (*n* = 17, 65.4%). The number of brain lesions per patient ranged from 4 to 10, with a median of [6–8 lesions]. Among these enrolled participants, several individuals (*n* = 9, 34.6%) received pyrotinib plus WBRT as their second-line therapy in the advanced stage. Furthermore, the rest of the participants (*n* = 14, 53.8%) received pyrotinib plus WBRT as their third-line therapy, while participants (*n* = 3, 11.5%) had already undergone at least fourth-line treatment with pyrotinib plus WBRT. Before their enrollment, some participants (*n* = 5, 19.2%) had not been administered any anti-HER2 therapy. In comparison between the WBRT-p and p-WBRT treatment groups, no statistically significant differences in the baseline characteristics were observed (Table 1; Supplementary material).

**Table 1:** A summary presents the patients’ characteristics.

	WBRT-p (*n* = 11)	p-WBRT (*n* = 15)	*p*-value
Age (years), *n* (%)	46.13 ± 3.12	50.27 ± 4.34	0.128
Menstrual state, *n* (%)			0.131
Premenopausal	9 (81.8)	8 (53.3)	
Menopausal	2 (18.2)	7 (46.7)	
ECOG performance status, *n* (%)			0.382
0–1	11 (100)	14 (93.3)	
≥2	0	1 (6.7)	
Hormone receptor status, *n* (%)			0.851
Negative	7 (63.6)	9 (60.0)	
Positive	4 (36.4)	6 (40.0)	
Histological grading, *n* (%)			0.741
II	8 (72.7)	10 (66.7)	
III	3 (27.3)	5 (33.3)	
Measurable lesions, *n* (%)			0.382
Intracranial lesions only	0	1 (6.7)	
Intracranial and extracranial lesions	11 (100)	14 (93.3)	
Site of extracranial metastases, *n* (%)			0.688
Viscera	2 (18.2)	5 (33.3)	
Bone and/or soft tissue	1 (9.1)	1 (6.7)	
Both	8 (72.7)	9 (60.0)	
Previous HER2-directed therapy, *n* (%)			0.430
Neoadjuvant or adjuvant therapy	4 (36.4)	5 (33.3)	
For advanced disease	2 (18.2)	6 (40.0)	
Both	3 (27.3)	1 (6.7)	
No	2 (18.2)	3 (20.0)	
Number of previous therapy lines in advanced setting, *n* (%)			0.067
1	6 (54.5)	3 (20.0)	
2	3 (27.3)	11 (73.3)	
≥3	2 (18.2)	1 (6.7)	
Prior brain metastases tx			0.635
None	2 (18.2)	4 (26.7)	
Radiation	5 (45.5)	8 (53.3)	
Surgery	4 (36.4)	3 (20.0)	
Baseline steroid use			0.951
Yes	6 (54.5)	8 (53.3)	
No	5 (45.5)	7 (46.7)	
Concurrent therapy with anti-HER2 drugs			0.722
Yes	8 (72.7)	9 (60.0)	
No	4 (36.4)	6 (40.0)	
No. of brain metastatic (*n*)			0.002
≤4	8 (72.73)	9 (60.00)	
>4	3 (27.27)	6 (40.00)	
Size of brain metastases			0.585
≤2 cm	4 (36.36)	3 (20.00)	
>2 cm	7 (63.64)	12 (80.00)	

WBRT-p, whole brain radiotherapy combined with concurrent or sequential pyrotinib; p-WBRT, pyrotinib combined with sequential whole brain radiotherapy; ECOG, Eastern Cooperative Oncology Group; HER2, human epidermal growth factor receptor 2.

### 3.2 Efficacy

**Figure 3:**
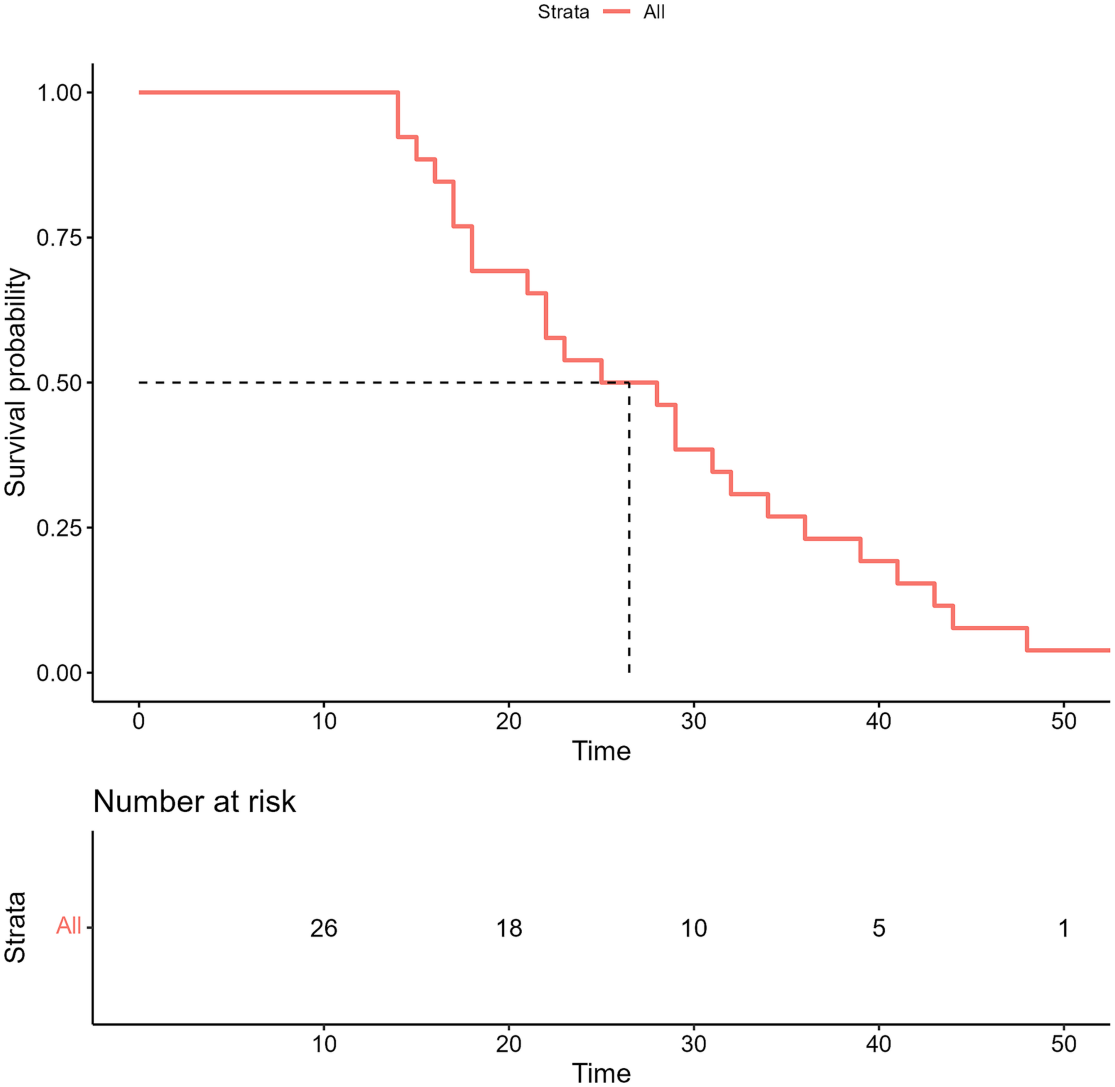
The image shows intracranial progression-free survival (iPFS) in all patients.

**Figure 4:**
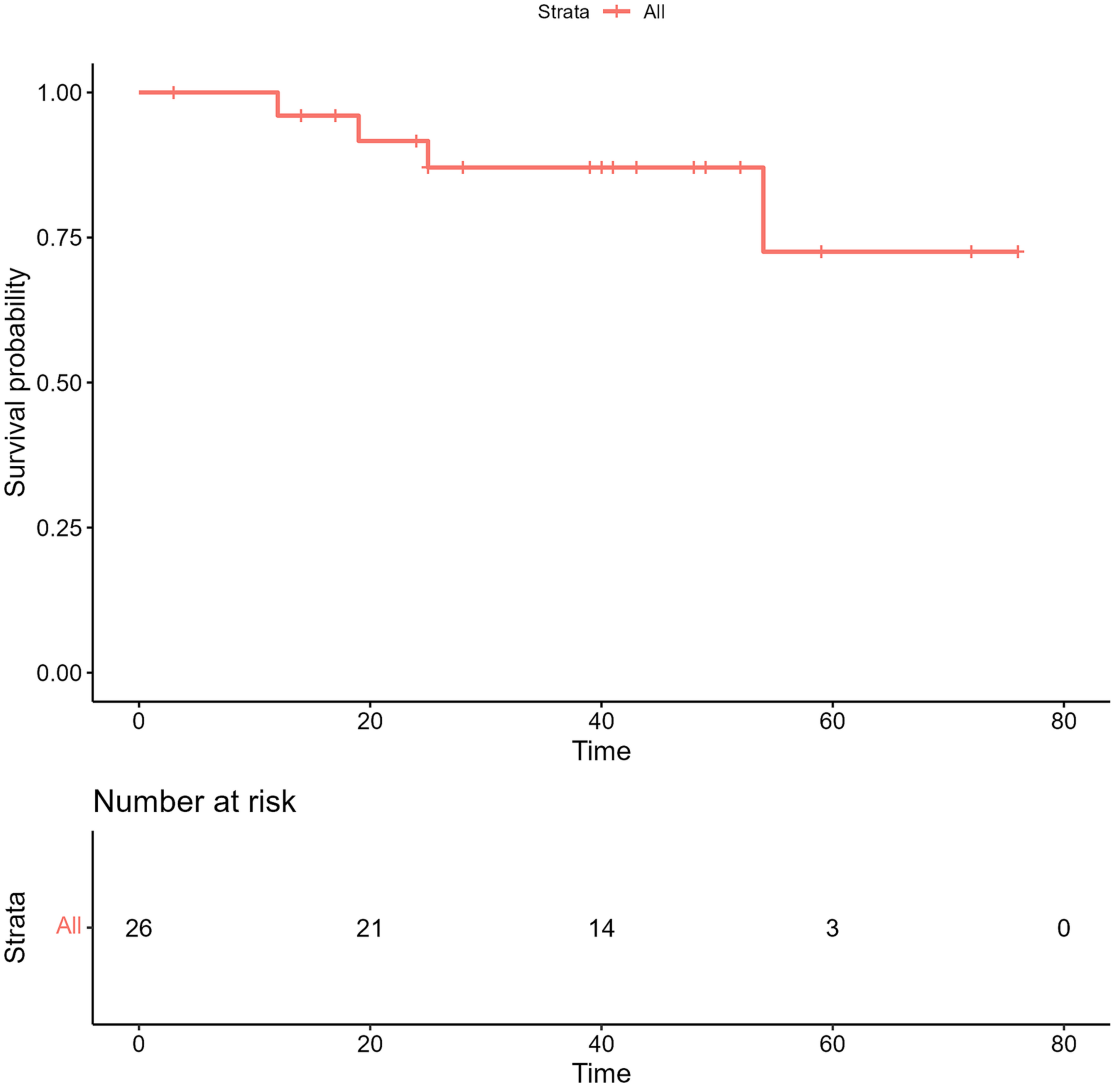
The image presents the overall survival (OS) in all patients. NR, not reached.

**Figure 5:**
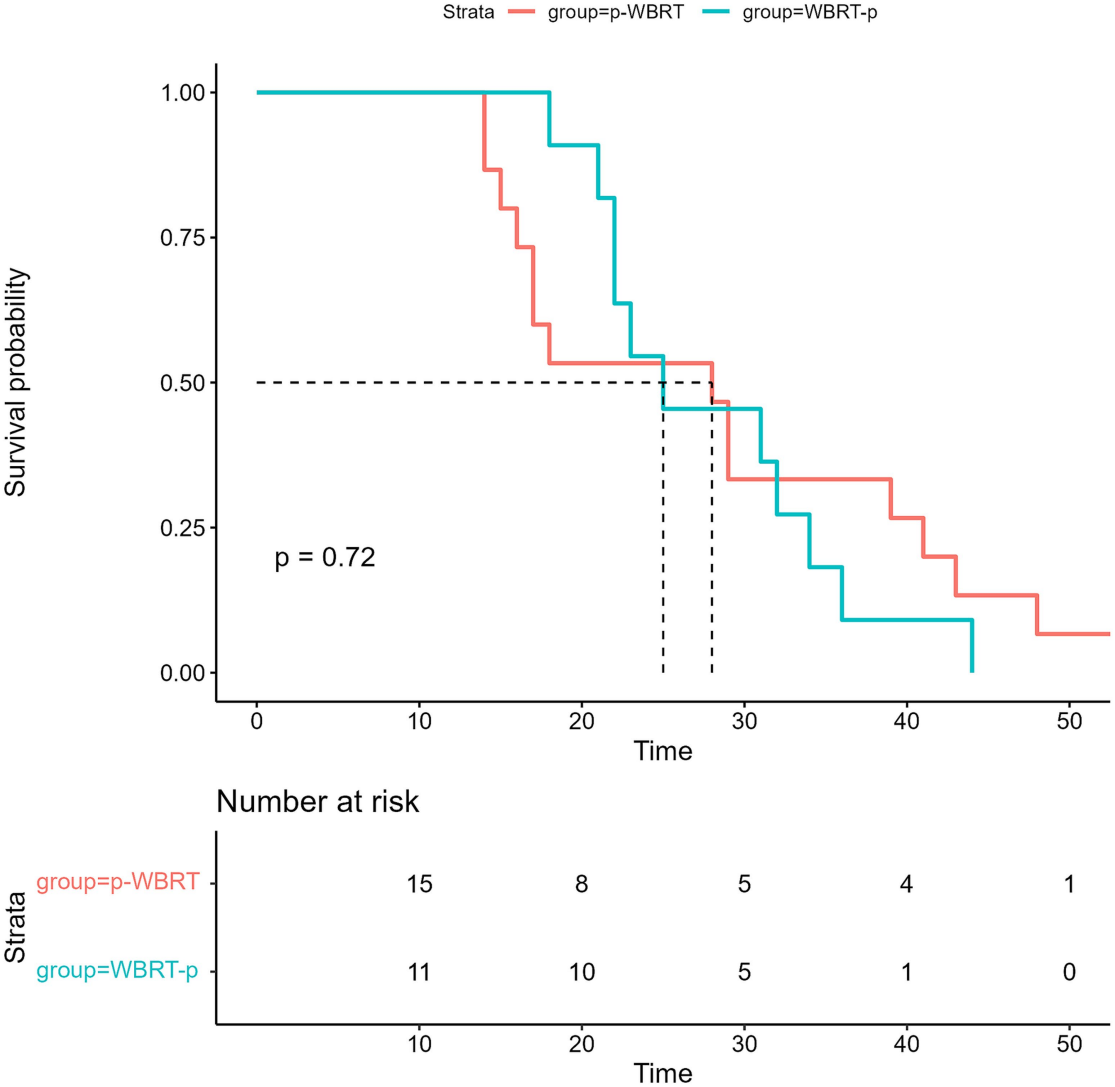
The image shows Intracranial progression-free survival (iPFS) in the WBRT-p and p-WBRT groups.

The median length of time for follow-up during this prospective study was 40 months. The median interval of progression-free survival (iPFS) was 25.0 months (95% confidence interval [CI], 18.0–32.0) (Figure 3), while the median OS for all the enrolled 26 patients was not met (Figure 4). However, no notable variation was observed in the recorded iPFS values (28.0 months [95% CI: 16.6–39.4] *vs*. 25.0 months [95% CI: 15.3–34.7], *p* = 0.72; Figure 5) between the groups that received WBRT-p and p-WBRT treatments. Within the WBRT-p group, the iPFS rate at 1 year was 100%, followed by 54.5% in 2 years, 9.1% in 3 years, and finally, 0% in 4 years. On the other hand, the p-WBRT group exhibited iPFS rates of 100% in 1 year, 53.3% in 2 years, 33.3% in 3 years, and 6.7% in 4 years.

**Figure 6:**
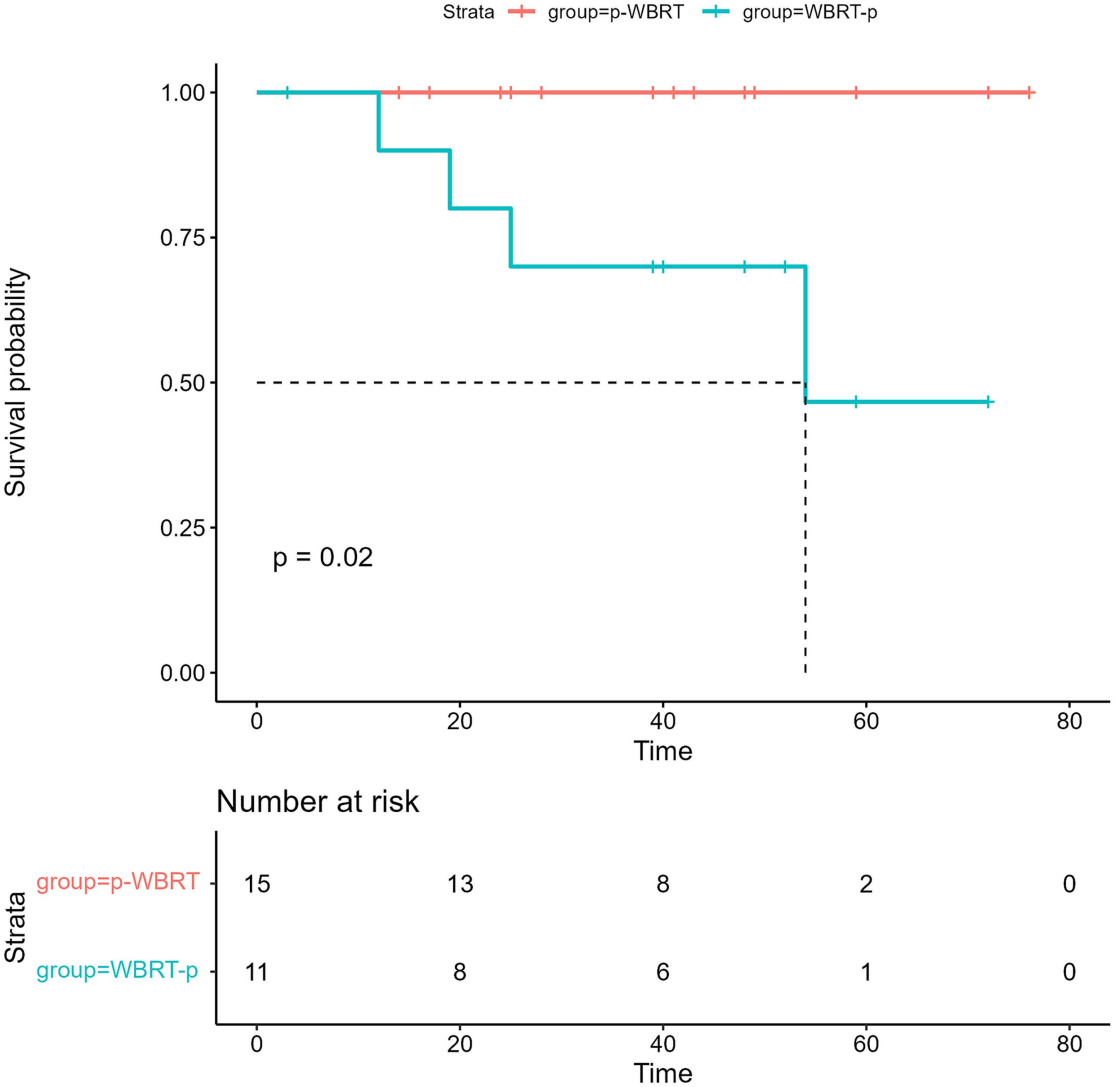
The image presents the overall survival (OS) in the WBRT-p and p-WBRT groups. NR, not reached.

**Figure 7:**
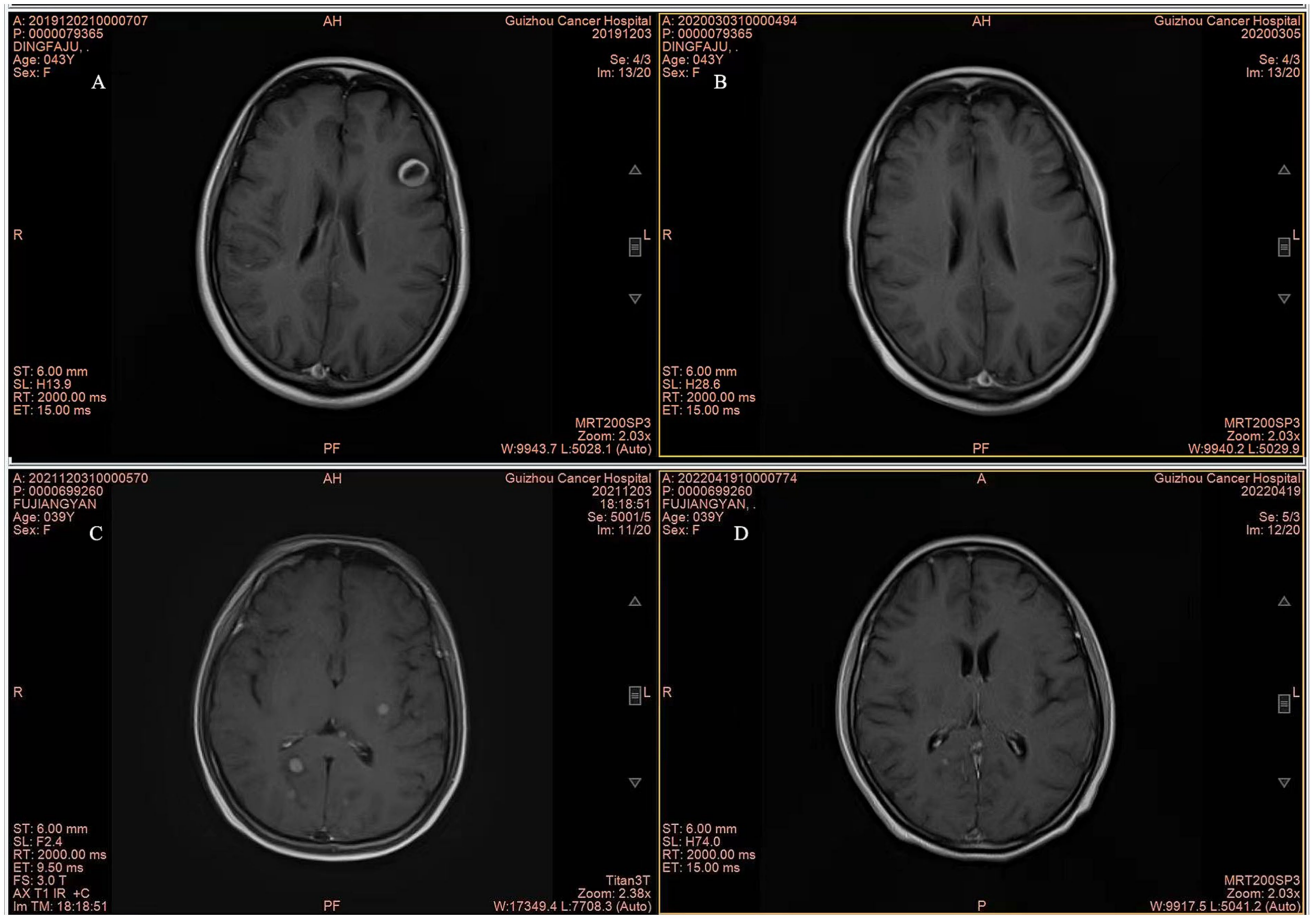
Representative MRI Images of patients. **(A,B)** MRI images show complete response (CR) in a patient from the p-WBRT group before and after treatment. The interval between the scans was 4 months. The images illustrate the complete disappearance of brain metastases over the course of treatment. **(C,D)** MRI images show partial response (PR) in a patient from the WBRT-p group, with a 4-month interval between scans. The images demonstrate a significant reduction in the size of brain metastases following the treatment.

Although the OS data remain immature, there was a significant difference in OS between the WBRT-p and p-WBRT groups (*p* = 0.02), with a trend of prolonged OS observed in the WBRT-p group (Figure 6). In the WBRT-p group, the OS rates in 1–5 years were 100, 100, 100, 100, and 100%, respectively, while in the p-WBRT group, the OS rates in 1–5 years were 90.0, 80.0, 70.0, 70.0, and 46.7%, respectively. For the overall population, the iORR value was 65.4% (17/26), and the iCBR value was 84.6% (22/26). Notably, no statistical significance was observed between the iORR and iCBR values in the WBRT-p group (63.6%, 7/11, and 90.9%, 10/11) and the p-WBRT group (66.7%, 10/15, and 80.0%, 12/15). As shown in the MRI images, a near-complete response (CR) with mild residual cortical enhancement was observed in the p-WBRT group, with scans taken 4 months apart (Figures 7A,B). Similarly, in the WBRT-p group, a partial response (PR) was observed, also with scans taken 4 months apart (Figures 7C,D). Regarding extracranial response, the extracranial ORR and CBR values were 69.2% (18/26) and 92.3% (24/26) in all 26 patients, 72.7% (8/11) and 90.9% (10/11) in the WBRT-p group, and 66.7% (10/15) and 93.3% (14/15) in the p-WBRT group, respectively (Table 2).

**Table 2:** A summary presents the treatment responses.

	WBRT-p (*n* = 11)	p-WBRT (*n* = 15)	Total (*n* = 26)	*p*-value
Intracranial response				
CR	0	1 (6.67)	1 (3.8)	
PR	7 (63.6)	9 (60.0)	16 (61.5)	
SD	3 (27.3)	2 (13.3)	5 (19.2)	
PD	0	3 (20.0)	3 (11.5)	
NE	1 (9.1)	0	1 (3.8)	
Intracranial ORR	7 (63.6)	10 (66.7)	17 (65.4)	1.000
Intracranial CBR	10 (90.9)	12 (80.0)	22 (84.6)	0.614
Extracranial response				
PR	8 (72.7)	10 (66.7)	18 (69.2)	
SD	2 (18.2)	4 (26.7)	6 (23.1)	
PD	0	1 (6.7)	1 (3.8)	
NE	1 (9.1)	0	1 (3.8)	
Extracranial ORR	8 (72.7)	10 (66.7)	18 (69.2)	1.000
Extracranial CBR	10 (90.9)	14 (93.3)	24 (92.3)	1.000

WBRT-p, whole brain radiotherapy combined with concurrent or sequential pyrotinib; p-WBRT, pyrotinib combined with sequential whole brain radiotherapy; CR, complete response; PR, partial response; SD, stable disease; PD, progressive disease; NE, not evaluable; ORR, objective response rate; CBR, clinical benefit rate.

### 3.3 Toxicity

Table 3 lists all of the patients’ TRAEs. Among the 26 patients who received treatment, no patients discontinued treatment due to severe side effects (SEs), and no dose modifications were required. In the WBRT-p group, a patient (1, 9.1%) had vomiting, with no patients suffering from cerebral necrosis and intracranial TRAEs. Contrarily, in the p-WBRT group, two patients (13.3%) experienced a decrease in white blood cell (WBC) and neutrophil counts. These patients had WBC counts between 3.0 and 2.0 × 10^9^/L and neutrophil counts between 1.5 and 1.0 × 10^9^/L. Treatment was not stopped, and the patients were given DiYu ShengBai tablets (0.4 g orally, three times daily) for observation. These hematological toxicities were reversible with supportive care, and all TRAEs were grade 1–3. No fatal or irreversible SEs were observed. Furthermore, all patients completed the planned treatment schedule without interruptions.

**Table 3:** A summary presents the treatment-related adverse events.

	WBRT-p (*n* = 11)	p-WBRT (*n* = 15)
Diarrhea	0	1 (6.7)
Anemia	0	0
Vomiting	1 (9.1)	0
Nausea	0	0
Alanine aminotransferase increased	0	0
Headache	0	0
Dizziness	0	0
White blood cells decreased	0	2 (13.3)
Neutrophil count decreased	0	2 (13.3)

WBRT-p, whole brain radiotherapy combined with concurrent or sequential pyrotinib; p-WBRT, pyrotinib combined with sequential whole brain radiotherapy.

## 4 Discussion

BC has emerged as the second most prevalent type of cancer, resulting in BM (18). Among the recorded cases, previous reports indicated that about 30% of individuals with advanced HER2+ BC might develop BM, significantly worsening their prognosis. The median survival rate for patients with BM is notably low, at around 5.9 months (19, 20). Despite the availability of treatment options, the outcomes for patients with BM remain poor, as reflected in a limited number of studies (21). Therefore, there is a pressing need for additional clinical research to explore and develop novel therapeutic approaches that could extend the survival rates of patients with BM.

In clinical practice, the general treatment modality for patients with HER2+ advanced BC and BM is local therapy combined with anti-HER2 systemic treatment. However, the absence of a standardized protocol is a limiting factor in achieving optimal treatment outcomes. SRS is an important alternative for patients with a smaller number of lesions. SRS offers targeted treatment with the potential to minimize neurocognitive side effects, and it is increasingly being considered for patients with up to 10 brain lesions, depending on the clinical context. In clinical practice, the choice between WBRT and SRS depends on several factors, including the number, size, and location of brain metastases, as well as the patient’s overall condition. While WBRT is often preferred for its ability to address both visible and microscopic disease throughout the brain, SRS is a valuable option for patients with fewer lesions, offering precise targeting and the potential for reduced neurocognitive impact. Therefore, the upfront WBRT is still the primary treatment approach for people with numerous brain lesions (22).

A series of studies of tyrosine kinase inhibitors (TKIs) have been developed for BM treatment in patients with HER2+ advanced BC. In a case, the phase-II PERMEATE study investigated the treatment with pyrotinib + capecitabine for patients with HER2+ metastatic BC and BM, with the primary endpoint being iORR (17). The results showed that the iORR with pyrotinib in conjunction with capecitabine in radiotherapy-naïve patients could reach 74.6%, indicating the promising value of pyrotinib combined with capecitabine for this population (1). The marked difference in iORR between radiotherapy-naïve patients (74.6%) and those who had previously received RT (42.1%) highlights the potential impact of prior radiotherapy on the efficacy of pyrotinib combined with capecitabine. This suggests that radiotherapy might compromise the response to subsequent systemic therapies, possibly due to changes in the tumor microenvironment or alterations in drug sensitivity induced by prior radiation. These findings underscore the importance of carefully considering the timing and sequence of treatments in managing HER2-positive metastatic breast cancer with brain metastases. In our study, we further explored the efficacy of combining WBRT with pyrotinib in patients with HER2+ advanced BC and BM. We divided the patients into two groups: one group received pyrotinib before WBRT (p-WBRT group), while the other group received pyrotinib either concurrently with or after WBRT (WBRT-p group). Our results showed that the median iPFS was 22.0 months in the p-WBRT group compared to 18.0 months in the WBRT-p group. Similar to the findings from the PERMEATE study, these results suggest that the timing of radiotherapy may significantly influence the effectiveness of subsequent systemic therapies like pyrotinib. Although our sample size was limited, and the statistical significance might not fully establish a clear difference between the two treatment approaches, the data suggest that administering pyrotinib before WBRT might help extend progression-free survival. Additionally, both treatment groups exhibited good safety profiles with no significant neurological damage or toxicity observed. However, it is noteworthy that the WBRT-p group experienced slightly fewer adverse effects, which could be related to the treatment sequence.

In the p-WBRT group, two patients (13.3%) experienced a decrease in WBC and neutrophil counts, which were managed without treatment interruption by administering DiYu ShengBai tablets. This mild hematological toxicity was reversible and did not lead to fatal outcomes. The slightly lower rate of adverse effects in the WBRT-p group may suggest a possible influence of treatment sequence on toxicity profiles. This observation indicates that administering WBRT prior to pyrotinib might help reduce certain side effects, although further studies with larger sample sizes are necessary to confirm this potential benefit. Clinically, the choice of treatment sequence could be tailored based on patient tolerance and baseline health status to optimize therapeutic outcomes and minimize side effects.

Additionally, the outcomes of two different treatment approaches were also compared in a retrospective study. These findings revealed no statistically significant difference in OS rates between the groups that received pyrotinib in combination with radiotherapy and pyrotinib-based therapy alone (23). Nonetheless, the median iPFS rate was numerically better with pyrotinib in combination with radiotherapy (15.0 months *vs.* 9.0 months). These findings suggested the feasibility of combining radiotherapy with anti-HER2 systemic therapy. Previous studies reported the ability of radiotherapy to induce the opening of the blood–brain barrier, thereby allowing increased drug concentration in brain tissue and cerebrospinal fluid (24, 25). Furthermore, it was reported that TKIs could enhance the sensitizing effect of radiotherapy, leading to greater antitumor efficacy (26). Accordingly, the combination of radiotherapy and TKIs showed the potential to improve the treatment outcome for certain diseases. Thus, radiotherapy combined with TKI might exhibit a synergistic antitumor effect. Although effective in treating various conditions, radiation therapy results in significant adverse effects on patients’ overall well-being. These associated shortcomings can greatly impair the quality of life for individuals undergoing treatment. Therefore, it is essential to cautiously consider the application of radiation therapy alongside the use of pyrotinib, establishing the best possible treatment plan for patients with BM. Considering these aspects, we ensure that the provided course of care can be effective, minimizing the negative impact on patients’ quality of life.

Previous investigation showed that the prognosis for patients treated with WBRT alone remains poor, with median survival typically ranging from 6 to 8 months and a significant drop in survival rates beyond 1 year. In a retrospective study, the effect of WBRT combined with pyrotinib and capecitabine was analyzed in 29 HER2+ advanced BC patients with BM. The findings suggested that the combination regimen of WBRT, pyrotinib, and capecitabine demonstrated exceptional therapeutic efficacy and was well-tolerated in the patient population. Moreover, the sequential utilization of pyrotinib and capecitabine with WBRT was found to be more practical compared to the concurrent use of pyrotinib and capecitabine with WBRT (27). Currently, pyrotinib, in combination with WBRT, is rarely used in patients with HER2+ advanced BC and BM. In our present work, the combination of pyrotinib with WBRT in our study demonstrated improved outcomes. Importantly, no patients discontinued treatment due to adverse effects, and no dose modifications were required, indicating a good overall safety profile for the treatment combination. Specifically, the 1-year overall survival rates in our study were 100% in the WBRT-p group and 86.7% in the p-WBRT group, suggesting an improvement over traditional WBRT outcomes.

This study demonstrated a favorable safety profile for the combination of WBRT and pyrotinib. No patients discontinued treatment due to treatment-related severe side effects (SEs). Observed TRAEs were consistent with the known safety profile of pyrotinib and radiotherapy, including mild to moderate vomiting and reversible hematological toxicities. These findings are in line with previous reports on pyrotinib and WBRT, which also documented manageable toxicities without significant long-term adverse effects. However, the slightly higher incidence of hematological toxicity in the p-WBRT group warrants further investigation to determine whether treatment sequence influences the toxicity profile. Importantly, the absence of treatment discontinuations or dose modifications underscores the tolerability of this regimen, which is crucial for patients with HER2+ advanced breast cancer and brain metastases. Future studies with larger sample sizes are needed to confirm these results and further evaluate rare or severe toxicities.

Although the small sample size might be the primary reason for the absence of a statistically meaningful distinction, both treatment strategies showed an excellent safety profile, with no significant neurological damage or toxicities. All the TRAEs reported in the study fit into grades 1–3, while the WBRT-p group appeared to show fewer adverse effects.

We acknowledge that SRS is generally preferred for patients with a small number of brain metastases to minimize neurocognitive side effects. However, in our patient population, all individuals presented with symptomatic brain metastases, which often necessitates more aggressive treatment. The decision to use WBRT was driven by the need to address the symptomatic and potentially widespread nature of the disease. Furthermore, our facility was limited in its ability to offer SRS, a situation that is not uncommon in many clinical settings. Despite these constraints, WBRT remains a rational choice for patients with multiple or symptomatic brain metastases, as it can provide broader intracranial control. Our study aims to explore the efficacy and safety of combining WBRT with pyrotinib under these real-world conditions, where such constraints may influence treatment decisions.

In our study, pyrotinib-assisted WBRT in patients with HER2+ advanced BC and BM showed promising efficacy with manageable toxicities. Compared to historical data for WBRT alone, where median survival typically ranges from 6 to 8 months with significantly lower 1-year survival rates (17–20%) (28), the combination of pyrotinib with WBRT in our study demonstrated improved outcomes.

This study further demonstrated a favorable safety profile for the WBRT and pyrotinib combination. No patients discontinued treatment due to treatment-related severe side effects (SEs), and no dose modifications were required. TRAEs included mild to moderate vomiting and reversible hematological toxicities, consistent with the known safety profiles of pyrotinib and WBRT. Although the p-WBRT group showed a slightly higher incidence of hematological toxicity, this warrants further investigation to determine whether treatment sequence influences toxicity profiles. Importantly, the absence of treatment discontinuations underscores the tolerability of this regimen, which is critical for patients with HER2+ advanced BC and BM. Despite the small sample size limiting statistical significance, both treatment strategies showed excellent safety profiles with no significant neurological damage or severe toxicities. All TRAEs reported were grade 1–3, and the WBRT-p group appeared to have fewer adverse effects. Larger studies are needed to validate these findings and to further explore the influence of treatment sequence on efficacy and safety.

This prospective study is limited by the lack of a standard control group. In actual clinical practice, it is difficult to set up a strict blank control or standard treatment control as in classic randomized controlled trials. The reason is that the patients with HER2-positive advanced breast cancer and brain metastases in our study had relatively complex and severe conditions. For ethical considerations, we could not withhold any active intervention measures from the patients. The prospective study also suffers from a shortcoming of a tiny sample size, resulting in the statistical insignificance between the two different treatment approaches. To determine the most appropriate timing for combining pyrotinib with radiotherapy, a larger trial will be necessary.

Furthermore, while our study did not reveal statistically significant differences in survival rates between the two treatment groups (p-WBRT and WBRT-p), there was a trend indicating that the sequence of pyrotinib administration relative to WBRT could influence progression-free survival (PFS). Specifically, the p-WBRT group, which received pyrotinib before WBRT, demonstrated a slightly longer median intracranial progression-free survival (iPFS) compared to that in the WBRT-p group. Although these differences did not reach statistical significance, likely due to the small sample size, they suggest that the timing of pyrotinib administration may impact clinical outcomes. Further research with larger sample sizes is necessary to validate these findings and to better understand the optimal sequence of treatments. During the initial screening phase, a small subset of patients did not meet the inclusion criteria or withdrew before starting the study treatment due to underlying clinical conditions or personal choice. These patients were excluded before enrollment and therefore were not part of the final cohort included in the analysis. No patients who began the study treatment discontinued due to adverse effects, and no dose modifications were required. Furthermore, the difference in the treat time between groups might impact the outcome of patients, therefore, further prospective study of large size sample including standard controls is needed.

## 5 Conclusion

In summary, this study based on the application of pyrotinib in combination with WBRT exhibited favorable outcomes in patients suffering from HER2+ advanced BC with BM, suggesting the treatment strategy could be an effective method in these patients.

## Data Availability

The data analyzed in this study is subject to the following licenses/restrictions: The data that support the findings of this study are available from at each participating facility but restrictions apply to the availability of these data, which were used under license for the current study, and so are not publicly available. Requests to access these datasets should be directed to liu_shu16@126.com.
